# Supplementary material for: Are carbon emissions trading and green financial instruments synergistic? -Comprehensive quantitative research based on content analysis
Source: PLoS One. 2024 Mar 7;19(3):e0298601. doi: 10.1371/journal.pone.0298601 (PMC10919601; doi:10.1371/journal.pone.0298601)
Supplement: S1 Data — (ZIP) [file pone.0298601.s001.zip › Data/地级市Z_F工作报告.zip!/░▓╗╒/║╧╖╩╩╨/└┤╫╘╣1⁄2╓┌║┼í╛┬φ┐╦╩2╛▌═°í┐_2015.docx]

# **2015年政府工作报告**

发布时间：2015-01-26 08:04

【字体：[大](http://www.hefei.gov.cn/public/1741/javascript:void(0);) [中](http://www.hefei.gov.cn/public/1741/javascript:void(0);) [小](http://www.hefei.gov.cn/public/1741/javascript:void(0);)】

各位代表：

   现在，我代表市人民政府向大会报告工作，请予审议，并请政协委员和其他列席人员提出意见。

   一、2014年工作回顾

   过去的一年，在省委省政府和市委的坚强领导下，我们全面贯彻落实党的十八大和十八届三中、四中全会精神，积极应对复杂多变的宏观环境，坚持稳中求进，突出改革引领，强化创新驱动，顽强拼搏，扎实工作，较好地完成了市十五届人大二次会议确定的主要目标任务，“大湖名城、创新高地”建设迈出坚实步伐、展示崭新形象。

   预计，全市生产总值5100亿元以上、增长10%左右；全社会固定资产投资5385.2亿元、增长18.1%；规模以上工业增加值2126.6亿元、增长12.3%；财政收入880.7亿元、增长14.6%，其中地方财政收入500.3亿元、增长14.1%；社会消费品零售总额1666.8亿元、增长12.9%；城镇常住居民人均可支配收入29500元、增长10%左右，农村常住居民人均可支配收入12700元、增长12%；城镇登记失业率3%；人口自然增长率7‰；居民消费价格涨幅2%；节能减排全面完成省控目标。

   （一）产业发展提质增效。以创新转型升级为导向，大力构建现代产业发展新体系。规上工业完成产值8447.8亿元，工业产销率达到96.7%。战略性新兴产业完成产值2553.9亿元、占全市工业30.2%，实现增加值681亿元、增长29.7%，对工业增长贡献率达到66.3%。国际智能语音产业园一期、高档数控装备研发基地、南车基地等重大项目开工建设，鑫晟8.5代线、欣奕华智能机器人、巨一自动化等建成投产，新型平板显示等电子信息产业、太阳能光伏等新能源产业分别增长49.7%、69.2%。推进“两化”深度融合，加快家电、汽车等传统优势产业改造升级，全年技改投入1172.9亿元、占工业投资的61.4%。出台现代服务业发展规划，获批国家电子商务示范市、信息惠民国家试点市、移动电子商务金融科技服务创新试点市、信息消费试点市、旅游标准化试点市。现代服务业完成投资1448.2亿元、增长20.4%。万达文化旅游城等加快建设，华南城一期等建成开业，国家数字出版基地入驻企业近300家，滨湖国际金融后台服务基地入驻机构增至15家。举办家博会等大型展会176场。房地产业平稳健康发展，“阳光地产”享誉全国。

   （二）改革攻坚激发活力。加强顶层设计，有序实施各项重点改革。出台扶持产业发展“1+3+5+N”政策体系，安排政策资金26.2亿元，首批市级政府投资引导基金实质性运转。深化全口径预算，实行政府债务计划管理和举债审批制度；深入推进政府向社会力量购买服务，累计实施159个项目，投入资金6.5亿元；“营改增”扩围试点顺利推进。创新重大项目和中小微企业融资模式，新增本外币各项贷款1246.6亿元，直接融资683.7亿元，应流机电、国祯环保、合锻机床首发上市，上市企业增至34家、居全国省会城市第7位。合肥三洋与惠而浦公司完成重组，合肥企业参与资本市场并购重组方兴未艾。注册资本、经营场所、“先照后证”等商事登记制度改革试点稳步开展，新增各类市场主体8.3万户、增长23.8%，民营经济对工业增长贡献率达到85.9%。制定加强土地管理进一步做好节约集约用地工作的政策意见，推动老城区存量建设用地升级改造，亿元生产总值消耗新增建设用地减至8.7亩、下降9.2%，完成全市集体所有土地确权登记。深化户籍管理制度改革，出台居住证管理办法。庐江县国家农业改革与建设试点等综合改革取得新进展，农村产权交易市场体系建设在全国首创新模式。基层医改任务全面落实，县级公立医院综合改革深入实施。工商、质监和食品药品监管体制改革顺利完成。供热等资源产品和环保价格改革有序推进。

   （三）扩大开放取得突破。积极适应对内对外开放新格局，大力打造全国内陆开放新高地。重大开放平台建设取得积极进展，合新欧国际货运班列实现常态化运行，合肥港二类水运开放口岸、合肥航空港进境水果指定口岸和B型保税物流中心成功获批，首条全货机航线实现通航，跨境电子商务产业园通关运行，综合保税区基础工程基本完成，出口加工区进出口额升至全国第7位。扎实开展第二批百名县干招商活动，深化与央企、知名民企、外资企业的合作发展，引进大项目96个，引资总量2950亿元、增长16%，其中外商直接投资21.8亿美元，境外世界500强企业增至37家。进出口总额达到200亿美元、增长35%。与乌法市等俄罗斯伏尔加河沿岸重要城市开展高层互访，与美国哥伦布、日本久留米、韩国原州等友城拓展合作领域。成功举办长江沿岸中心城市经济协调会第十六届市长联席会议。合肥经济圈一体化加快推进，与皖北结对合作扎实开展，临泉庐阳现代产业园获批设立，阜阳合肥现代产业园区、寿县蜀山现代产业园建设取得新进展。援疆、援藏工作取得新成效。

   （四）自主创新成效显著。大力实施创新驱动战略，加快合芜蚌自主创新综合试验区和国家创新型试点城市建设。全社会研发投入占生产总值比重达3.1%、居全国省会城市第5位，发明专利申请量达12929件、居全国省会城市第6位。科研能力大幅提升，获得年度国家科学技术奖9项，中科院合肥大科学研究中心获批筹建。协同创新平台加快建设，中科大先进技术研究院共建研发中心33个、孵化创新企业74家，清华大学合肥公共安全研究院、合工大智能制造技术研究院、中科院合肥技术创新工程院、北大未名生物经济研究院全面开建，现代显示等十大战略性新兴产业研究院转化科技成果114项。

   创新主体实力增强，新建工程中心等研发机构139家，新建院士工作站5家、在肥工作院士达到72人；新认定国家级高新技术企业373户，总数增至828户，居全国省会城市第8位。进入国家首批科技成果使用、处置和收益管理改革试点，开展股权和分红激励试点企业增至147家。科技与金融深度融合，设立天使投资基金，开发科技创新贷、科技保险等金融产品，新三板挂牌企业达17家。

   （五）城市功能日益完善。以“1331”空间发展战略为引领，提升城市规划建设管理水平。四个城区加快老城区改造提升和都市产业园转型发展，打造全省“首善之区”和辐射源取得新成效。四大开发区坚持产城一体，工业发展主引擎、创新发展新高地的作用不断强化。滨湖新区区域金融、文化、旅游中心功能逐步显现。大建设全面推进，新建、续建工程667项，完成投资363.5亿元、增长28.2%。高铁南站及铁路枢纽南环线建成运行，合福高铁加快建设，火车西站改造启动实施，庐铜铁路开工建设，商合杭、合安九等高铁项目前期工作进展顺利。合肥港综合码头二期工程主体完工，合裕线航道、店埠河航道升级改造加快推进。合六路、合马路等全面完工，环巢湖公路全线贯通，“一环八线”国省干线公路网基本形成、一级公路总里程增至420公里，国省干线公路建设成为全国样板。城市轨道交通第二轮建设规划获批，1、2号线建设加快推进，3号线开工建设。郎溪路立交、龙川路、黄山路东延等建成通车，“畅通二环”、望江路改造等加快推进。新增、更新公交车辆1000台，入选国家公共交通智能化应用示范城市；水电气热等公用设施进一步完善。出台社会信用体系建设规划，颁布城市管理条例和餐厨垃圾管理办法，“数字城管”工程建成运行，违法建设和非法、到期户外广告依法全面拆除，“垃圾乱扔、摊点乱摆、车辆乱停”等专项整治深入开展，争创全国文明城市成效显著。

   （六）城乡统筹深入推进。以“工业发展主战场、城乡统筹新典范”为统揽，推进城乡一体融合发展。加大对五县（市）政策、资金等支持力度，县域经济总量达1700亿元、占全市33.6%，县域规上工业增加值786.7亿元、占全市37%。肥西在全国百强县位次升至第86位，肥东再度跻身全国科学发展百强县，巢湖、长丰获评全省美好乡村建设先进县，庐江入围全省农村金融改革试点县。粮食生产实现“十一连丰”，肉蛋奶水产品总产量达到102.8万吨，新建“粮安工程”仓容40.5万吨。市级以上特色农业园区超过400个，省级现代农业示范区在全省率先实现县域全覆盖。新增专业大户500多个、家庭农场750多个、农民合作社500多个，农业产业化联合体经营模式引领全省。董铺和大房郢水库水源地、新桥国际机场周边、庐江汤池三大土地整治项目完成农田整治21万亩、村庄整治2万亩。耕地连续16年实现占补平衡。积极推进与国开行合作新型城镇化一期试点项目建设，县城、新市镇、环湖十二镇等规划建设取得新进展，首批49个美好乡村重点示范村通过省级验收，第二批82个重点示范村和656个自然村整治深入推进，以“三线三边”为重点的全市域环境综合整治取得实效。

   （七）生态建设不断加强。强化生态文明理念，统筹推进环境保护和节能减排。巢湖流域跻身首批国家生态文明先行示范区。与国开行合作的环巢湖地区生态保护修复工程一期全部竣工，二期全面开工，三期获批开贷。严格落实“河长制”，强力推进南淝河、十五里河、派河综合治理，望塘污水处理厂PPP试点有序推进，王小郢污水处理厂提标改造如期完成，环湖32座乡镇污水处理厂建成调试，巢湖流域11个国考断面7个达标、创历史最好水平。完成植树造林25.6万亩、城区绿化1410万平方米，全市森林覆盖率达28.6%，成功创建国家森林城市。实施大气污染防治“九大行动”，加大扬尘治理力度，首次全市域午秋两季秸秆禁烧成效显著，完成淘汰黄标公交车和市级机关事业单位黄标公务车任务；“气化合肥”深入推进，新增LNG汽车600辆、CNG汽车7000辆；全年PM10浓度下降1.7%，空气质量优良天数190天，比2013年增加8天。

   （八）社会建设全面推进。以编制规划和加大投入为重点，不断提升基本公共服务水平。各级财政投入71.8亿元，全面实施“32+14”项民生工程，民生方面总支出552.2亿元、占财政总支出79%。深入实施就业促进工程，新增城镇就业18.9万人，转移农村劳动力12.5万人。完善社会保障体系，新农合筹资标准增加到年人均390元，大病保险实现全覆盖；农村低保提标到年人均1850元以上，散居五保对象供养标准提高到年人均2420元；开工各类保障性住房和棚户区改造安置房4.8万套（户）。建立精准扶贫机制，“光伏扶贫”模式在全国推广；7个“老字号”群体生活补助实现全覆盖；“渔民上岸”安居工程提前完成。完善临时价格补贴联动机制，肉类蔬菜流通追溯体系试点建设领先全国。实施118个老旧小区环境综合整治，惠及居民5.7万户、14.9万人；实施城中村和棚户区改造30个，完成房屋征收面积近300万平方米，受益群众1.9万户、5.7万人。学前教育改革试点顺利推进，新建、改扩建公办幼儿园29所，新增普惠性民办幼儿园35所；义务教育“三大提升工程”深入实施，学校标准化建设完成率达92%，义务教育均衡发展通过国家认定的县（市）区增至7个，进城务工人员随迁子女定点学校增至206所；普通高中教育、职业教育、高等教育质量进一步提升，合肥学院荣获国家级教学成果一等奖。建成7个省级和50个市级农民文化乐园，安徽名人馆即将开馆，“全民文化周”、“悦读合肥”等活动深入开展。市儿童医院开工建设，基层医疗机构建设全部达标。旅游、文化、体育等融合发展，成功举办首届环巢湖国际马拉松赛，在省第十三届运动会上夺得六项第一。“单独二孩”政策有序实施，计划生育“后进村”转化率达95%。食品药品重大事故“零发生”，油气管线隐患专项整治全国领先，各类安全生产事故指标创历史最低水平。“天网工程”全面建成，治安防控体系进一步完善；“信访积案化解绩效年”活动取得实效，信访渠道进一步畅通，法治合肥和平安合肥建设不断深化。民防进社区深入推进，人防基础设施建设不断强化，气象监测预警工程建成使用，地震活断层探测项目通过中期评估，应急处置能力进一步提升。坚持军民融合深度发展，国防动员和后备力量建设扎实推进。《走读合肥》丛书成功发行，《合肥通史》编纂取得阶段性成果，二轮修志全面完成。民族宗教、新闻、科普、统计、档案、保密等工作水平实现新提升，工会、共青团、妇女儿童、红十字会、残疾人、老年人和关心下一代等工作取得新进步。

   各位代表！

   过去的一年，我们深入开展党的群众路线教育实践活动，大力整治“四风”，切实解决群众反映强烈的突出问题，政府作风建设取得明显成效。严格执行中央“八项规定”、国务院“约法三章”和省市有关规定，各类会议、文件和检查、评比大幅精简，“三公经费”支出同比减少20.6%，党政干部因公出国人数同比下降11.6%。完善政府决策程序，出台重大行政决策听证、风险评估、实施效果评估等3个办法，强化重点领域和关键环节的监察、审计。大力精简行政审批事项，保留134项，减少169项，精简率达55.8%，成为全国审批事项最少的省会城市之一。开展第二届政府工作创新奖评选，10个项目获奖。坚持依法行政，严格执行人大及其常委会的决议决定，自觉接受人大监督；推进协商民主，主动接受政协民主监督，广泛听取民主党派、工商联、无党派人士和人民团体意见；虚心接受社会舆论监督。全年办理人大代表议案和建议243件、政协提案673件，办理质量和满意度大幅提升。

   各位代表！

   过去的一年，我们审时度势、超前谋划、精准施策，主要经济指标保持在“两位数”的较快增长区间内，经济总量跃上新台阶，“新跨越、进十强”的目标逐步实现，成绩来之不易。这是省委省政府和市委坚强领导的结果，是市人大、市政协和社会各界大力支持的结果，是全市广大干部群众团结奋斗的结果。在此，我谨代表市人民政府，向全市广大工人、农民、知识分子、干部、驻肥解放军指战员、武警官兵和政法干警，向各民主党派、工商联、人民团体和社会各界人士，向所有关心支持合肥建设和发展的海内外朋友，表示衷心的感谢，并致以崇高的敬意！

   在肯定成绩的同时，我们也清醒地看到存在的问题：经济下行压力依然较大，企业投资意愿下降，市场消费需求不旺，部分行业和企业生产经营困难，全市生产总值、规上工业增加值两项指标与预期目标尚有差距；市场监管、征地拆迁、信访维稳等矛盾纠纷增多，社会治理难度加大；生态、资源与环境约束趋紧，大气污染防治形势严峻，巢湖综合治理任务艰巨；个别部门和少数工作人员效能建设出现反弹，“四风”问题和庸懒散现象还不同程度存在，特别是思想观念、工作方式等还不能完全适应新形势新任务的要求。对此，我们一定高度重视，采取切实有效措施加以解决。

   二、2015年形势和任务

   今年是全面完成“十二五”规划的收官之年，是全面深化改革的攻坚之年，也是全面推进依法治国的开局之年，做好今年工作意义重大。当前，世界经济处于缓慢复苏和格局重塑的新时期，我国经济进入增长动力重构和发展方式转换的新常态，我市经济既要保持中高速增长，又要向中高端水平迈进。合肥发展的外部环境和条件正在发生深刻变化，但仍然处于大有可为的黄金发展期；世界科技革命和产业变革正孕育着新的重大突破，我们拥有的条件更好、基础更牢，面临的机遇更大、起点更高。国家大力实施新的区域发展战略，特别是推进长江经济带建设，明确把合肥定位为长三角世界级城市群副中心和全国性综合交通枢纽，首次要求合肥提升都市区国际化水平、建设全国内陆经济开放高地，我市在全国区域发展新棋局中的战略地位更加凸显。省委省政府全力支持合肥打造“大湖名城、创新高地”，要求合肥在更高的起点上谋划推动改革发展，当好全省“三个排头兵”，进一步增强辐射力、带动力、影响力，为合肥加快建设和发展注入了强大动力。我们要充分认清新形势、主动适应新常态、积极抢抓新机遇，坚定信心、奋发有为、乘势而上，全力把合肥建设和发展推上更高境界。

   今年政府工作的总体要求是，深入贯彻落实党的十八大和十八届三中、四中全会精神，在省委省政府和市委的坚强领导下，坚持稳中求进、稳中强基、稳中创优，强化改革攻坚，强化开放集聚，强化创新驱动，强化民生保障，解放思想，防骄破满，拉高标杆，跨越赶超，保持经济较快发展和社会和谐稳定，主要经济指标稳固“进十强”，全面完成“十二五”发展目标，为打造长三角世界级城市群副中心奠定坚实基础，进一步开创建设“大湖名城、创新高地”的新局面。

   今年经济社会发展的预期目标是：全市生产总值增长9.5%左右；财政收入增长9.5%，其中地方财政收入增长8%；全社会固定资产投资增长15%左右；规模以上工业增加值增长11%以上；社会消费品零售总额增长12%；居民人均可支配收入增长10%，城镇常住居民人均可支配收入增长9.5%，农村常住居民人均可支配收入增长10.5%；城镇登记失业率控制在4.5%以内；居民消费价格涨幅控制在3%以内；人口自然增长率保持在7.5‰以内；单位生产总值能耗下降率及主要污染物减排量达到省控目标。

   今年，着力做好八个方面工作：

   （一）加快产业转型升级

   瞄准国际国内前沿和高端，以创新转型升级为导向，突出重点，聚焦发力，加快打造若干全国性乃至世界级的产业集群。

   培育壮大新兴产业。在继续推进十大重点产业发展的同时，进一步聚焦关键核心技术，实施一批居于产业链核心环节的重大项目，加快产业转型升级。以京东方等为龙头，推进高世代面板生产线等建设，加快建设国际一流的新型显示产业集聚区。实行设计、制造、封装测试同步推进，加快建设IC之都。推进国际智能语音产业园建设，积极发展各类软件产业，加快打造“中国声谷”和全国软件名城。推进量子通信京沪干线等建设，加快创建引领全国的未来网产业基地。大力发展工业和服务机器人，加快建设全国性智能制造产业集聚区。大力推进燃气轮机、精密制造等技术产业化和数控装备发展，规划建设国家强基技术产业化基地。开发储能、微网及智慧能源等新技术，积极探索分布式光伏电站建设运营新模式，推进建筑光伏一体化发展，大力实施“光伏下乡”、“光伏扶贫”等工程，加快打造“中国光伏应用第一城”。以纯电驱动为技术路径，以落实和完善政策为主攻方向，集中力量建设和运营好一批充电设施，推进新能源汽车研发、示范应用和产业化。大力发展生物医药产业和医疗装备材料产业。坚持分类指导，整体推进四个城区、四大开发区和五县（市）转型发展。围绕重大领域、重量产业、重点项目和重要园区，深化开发性金融合作，推进产城一体，实现新兴产业全产业链发展。

   改造提升传统产业。坚持以高新化为导向，按照工业4.0思路，积极运用信息化和工业化深度融合的核心技术，推行智能制造和智慧管理，加快家电、汽车、装备制造等传统优势产业改造升级，建设一批智能工厂，全年技改投入达到1160亿元。大力实施标准化、品牌化战略，积极扶持龙头企业，推动企业加速向产业链两端延伸、价值链高端提升。启动“企业诊断”对标行动，鼓励企业按照产业发展方向加大投入力度，不断提高产品结构层次。加强经济运行调节，加大困难企业帮扶力度。加快推进瑶海全国老工业区整体搬迁改造试点。以减量化、资源化、再利用为方向，推进静脉产业基地规划建设和循环经济发展。

   大力发展现代服务业。突出发展工业设计、检验检测、信息技术、科技成果转化、数字内容等新型服务业态，着力打造国家级高技术服务业基地。大力发展电子商务、跨境电子商务、移动电子商务、服务外包等，推进蜀山国际电商产业园等专业园区建设，完善滨湖国际金融后台服务基地功能。发展文化创意产业，推动文化、体育、旅游等融合发展，大力实施万达文化旅游城等重大项目，积极支持三河古镇创建国家5A景区，扎实推进全国旅游标准化试点市建设，加快打造环巢湖国际旅游目的地。组建在肥基地航空公司，积极发展航空产业。高标准建设中央商务区，着力打造一批特色街区和规范有序的摊群夜市，构建市民“一小时消费圈”和“10分钟生活圈”。稳定住房、汽车、电子信息等大宗消费，重点培育家政服务、康复养老、休闲度假、体育健身、高端医疗等服务性消费，鼓励引导传统服务业利用互联网、物联网、大数据、云计算等信息技术实现提档升级。策划举办一批重大展会活动，拉动城乡消费增长。

   加快建设现代农业。全面实施现代农业建设“十大行动”，建设一批优势突出、特色鲜明的农业“块状经济”集中区。大力推进现代农业示范区建设，做大做强7个省级以上现代农业示范区主阵地，联动推进各类农业园区建设。加快发展新兴农业产业，在全国率先建设安全农产品加工园区，着力打造全国现代种业强市。坚持发展生态农业与巢湖面源污染治理相结合，促进农业可持续发展，在环湖乡镇开展化肥、农药减量化使用试点。加强与全国、全省农业科研机构合作，深入实施农业重大技术推广和科技入园入户工程，大力推进农业科技企业孵化基地建设。进一步扩大规模，规范运作，规避风险，稳定收益，推动土地流转扎实有序进行，鼓励引导农业适度规模经营。

   （二）大力实施改革攻坚

   围绕打造先行先试“合肥版”，按照“四个率先”的思路，推进重点领域和关键环节改革攻坚突破，营造体制机制新优势。

   加快经济体制改革。深化国有企业改革，推进股权多元化，完善产权管理、法人治理等制度，大力发展混合所有制经济。构建科学合理的国有资产运营监管体系，实现国有资产监管全覆盖。加强中小微企业服务体系建设，创新财政金融产品，推进新型政银担合作试点，完善小额担保贷款、政策性再担保和上市、挂牌等融资支持政策，优化投资环境，加快民营经济发展。深化预算管理制度改革，完善政府预算体系，扩大预决算公开范围；进一步完善扶持产业发展“1+3+5+N”政策体系，清理规范税收和财政支出等优惠政策，落实“营改增”等结构性减税政策；探索中期财政规划管理，全面推进财政绩效管理；推进国库集中支付电子化，激活财政存量资金，推行专项资金竞争性分配。加快建设移动电子商务金融科技服务创新试点市，积极推动设立民营银行、金融租赁公司、金融消费公司等新型金融机构。进一步推进土地管理制度改革，拓展节约集约用地试点。深化价格改革，完善市场决定价格机制。加快城乡一体化综合配套改革试验区建设，全面推进农村产权交易市场体系建设，推动城乡要素合理流动。

   加快行政体制改革。深化行政审批制度改革，扎实做好国务院、省政府取消下放审批事项的承接和落实，全面推行权力清单、责任清单、涉企收费清单制度，完善事中事后监管机制。加强电子政务平台建设，广泛推行网上审批。深入推进市场监管体制“三合一”改革，加快建立权责统一、权威高效的行政执法体制。继续深化商事制度改革，全面推进“先照后证”和“三证合一”改革。深化投融资体制改革，完善政府债务管理制度体系，积极推进PPP模式在城市基础设施和公共服务领域的应用。深化公共资源交易管理体制改革，全力推进省市平台共建，加快形成区域性要素大市场。创新公务员管理制度，推行公开遴选新机制，开展聘任制公务员试点。完成公车制度改革任务。加快事业单位分类改革，全面完成承担行政职能事业单位和生产经营类事业单位改革。

   加快社会体制改革。理顺市、区、街道、社区管理职能，推进社会治理模式创新，完善社会治理体系，大力培育社会组织，壮大志愿者队伍。进一步健全促进就业机制，深化收入分配制度改革。完善社会保障制度，稳妥推进机关事业单位养老保险制度改革，加快建立统一的城乡居民基本医疗保障和养老保险制度。深化教育领域综合改革，建立具有合肥特色的中小学质量评价指标体系。实行医疗、医保、医药改革联动，巩固完善基层医改成果，全面深化县级公立医院改革。推进文化体制改革，大力构建现代公共文化服务体系，加快发展文化产业。进一步完善廉租住房与公租房并轨运行、分类补贴和梯度保障机制，鼓励社会资本参与保障房建设。在食品药品安全、环境保护、安全生产、债券发行、招投标等领域开展信用体系建设试点，加快构建覆盖全社会的信用信息共享服务平台。

   （三）扩大对外开放合作

   坚持引资与引智并举、“引进来”和“走出去”并重，拓宽对外开放渠道，提高对外开放层次，全力打造全国内陆开放新高地。

   加快开放平台建设。全面建成综合保税区并按时封关运行，不断提升出口加工区层次和水平，高标准建设保税物流中心、合肥航空港进境水果指定口岸、合肥国际内陆港、跨境电子商务产业园二期、对外劳务合作服务中心等，推动合新欧国际货运班列西延入欧、国际货运航班直达欧美。积极推进长三角大通关建设和长江经济带海关区域通关一体化发展，主动对接上海自贸区，加快形成与国际投资、贸易通行规则相衔接的制度体系。

   推进对外合作发展。深化中美绿色合作伙伴关系，继续推进与俄罗斯伏尔加河沿岸联邦区合作，积极开展与更多的国际友城结好。认真筹办长江中游城市群省会城市第三届会商会议，积极参加长三角第十五次市长联席会议。推动合肥经济圈交通建设、产业布局、环境治理等一体化发展，全力支持皖北发展，加快推动阜阳合肥现代产业园区、寿县蜀山现代产业园做大做强，启动建设临泉庐阳现代产业园。积极实施“走出去”战略，完善外经外贸政策，引导优势企业通过绿地投资、并购投资、证券投资、联合投资等方式，开展跨国并购重组，开拓国际市场。

   强化招商引资工作。进一步发挥产业基金、风险基金的投资引导作用，依法依规完善招商政策体系，重点推进战略性新兴产业和现代服务业招商引资，着力提高项目质量和层次，更加注重引才引智。积极支持“合肥之友”拓展综合性、国际化平台功能，充分发挥行业协会、商会组织、高校校友会等中介作用，扎实开展百名县干招商活动，继续深化与世界500强、中国500强以及央企、知名民企等“点对点”、“一对一”合作，大力实施以商招商、小分队招商、登门招商和平台招商。全年完成招商引资3390亿元、增长15%，其中外商直接投资25亿美元。

   （四）提高自主创新能力

   以平台为支撑，以企业为主体，深入实施创新驱动战略，大力打造全国创新资源高度集聚区和国家自主创新示范市。

   完善协同创新平台。合理规划科技布局，形成功能强大的协同创新平台体系。大力推进中科大先进技术研究院管理运行机制创新，增强转化、集聚和辐射功能；加快建设清华大学合肥公共安全研究院、合工大智能制造技术研究院、中科院合肥技术创新工程院、北大未名生物经济研究院和十大新兴产业研究院，实施清华启迪科技城、数控装备研发基地、智能家居研发设计中心等一批重点项目。积极深化与中科院、在京中央高校以及国内其他知名高校、院所合作，加强与中关村等科技园区对接。加快建设科技企业孵化器，大力发展知识楼宇、科技楼宇，着力打造科技服务业密集区。承办好机器人世界杯大赛和中国计算机大会。

   增强企业创新能力。推进企业技术创新体系建设，构建企业主导的政产学研用协同创新机制。新增各类研发机构100家以上、国家级高新技术企业100家以上，全市高新技术和创新型企业总数达2000家以上，争创一批国家级、省级研发机构。开展市级工业设计中心认定，加快工业设计人才队伍和中国（合肥）工业设计城建设。支持现代显示、光伏、新能源汽车等重大研发平台建设，在集成电路、生物医药、智能制造、智慧城市等领域组建一批技术创新战略联盟。加大企业基础研究投入，鼓励社会资金和政府基金合作，形成社会共担机制。

   优化创新发展环境。加快改革科技成果产权制度、收益分配制度和转化机制，进一步放大企业股权分红和激励试点效应。强化知识产权保护，推进“中国（安徽）知识产权维权援助中心”建设。规划建设科创社区，创新人才聚集政策。发挥天使投资基金等引导作用，通过种子基金、风险投资等方式，对科技创新实行普惠式、引领式扶持。改革人才评价体系，不拘一格用好人才。鼓励高校、科研院所在海外设立人才招引工作站，加快引入一批高层次科技人才团队。支持培养本土人才，鼓励草根创新、蓝领创新和青少年创新。降低创业门槛，优化创业环境，完善创业辅导，强化创业扶持，积极引导和鼓励高校学生、科研人员等大众创业。在全社会大力弘扬创新创业文化，营造鼓励探索、宽容失败和尊重劳动、尊重知识、尊重人才、尊重创造的浓厚氛围。

   （五）加强城市规划建设管理

   坚持规划引领，推进全国性综合交通枢纽建设，提升都市区国际化水平，积极打造长三角世界级城市群副中心。

   加强重大规划编制。加快推进“四规合一”，完成全国性综合交通枢纽、市政基础设施、都市区国际化等重大规划编制，努力实现主城区控制性单元规划全覆盖。进一步完善新型城镇化规划，调整优化土地利用总体规划，加快形成“中心城-副中心-新市镇-新社区”的市域城镇等级体系。

   建设综合交通枢纽。启动新桥国际机场二期工程前期工作，统筹谋划老机场利用和通用机场建设。开工建设商合杭、合安九高铁，加快建设宁西复线、庐铜铁路，建成合福高铁并投入使用，开展与周边中心城市铁路加密的前期谋划，完善合肥铁路枢纽功能。加快引江济淮项目前期工作，力争早日开工；推动合裕线、兆西河、店埠河、丰乐河等航道升级改造。加快合安、合淮、合水、军二、合铜、巢庐、新合蚌路、合六南通道等国省干道改造建设，推进3条机场应急通道和4条环巢湖公路连接线建设。实施北沿江、滁新高速公路合肥段建设，加快合宁、合安、合巢芜高速扩容前期工作。

   完善城市承载功能。推进城市轨道交通工程建设，1号线实现隧道贯通、轨道铺通，2号线主体土建工程基本完成，3号线全面开建，加快4、5号线前期工作。完善城市路网体系，全面推进魏武路、“畅通二环”等重点项目建设，完成繁华大道东延、临泉东路等续建项目建设；继续推进支路网建设和小街巷改造，完善慢行系统。大力推进老城区停车场和电动汽车充电桩等规划建设，启动全国公交智能化应用示范工程，推进公交专用道建设，新增和更新公交车600台。加快改造城区燃气、供水、供热老旧管网，实施综合管廊建设试点，全面推进七水厂二期、八水厂、绕城高压环网输气干线、金源热电联产机组改扩建、滨湖新区区域供冷供热等项目。落实电力体制改革试点方案，实施中长期能源战略，推进华能巢湖电厂二期、神皖庐江电厂等项目建设。强化电网保障能力，确保供电安全有效。

   提升城市管理水平。构建“智慧合肥”建设总体框架，加快形成信息资源互联互通、共建共享的统一平台。完善“数字城管”工程运行机制，优化城市管理网格流程。健全“三城同创”长效机制，提升城市文明指数。以扁平化为方向、信息化为手段、网格化为基础，提高城区街道社区管理和服务的精细化、专业化水平。完善合肥南站综合管理体制，并推广到合肥站地区。加强城市立面景观综合整治，加大违法建设等查处力度，积极推进非法营运联合治理，全面规范道路交通管理秩序。开建小仓房大型生活垃圾中转站，推进餐厨垃圾无害化处理，严控固废和噪音污染。

   （六）加快城乡一体发展

   加大统筹力度，推进城乡联动发展、融合发展，加快建设全省乃至全国城乡一体化发展示范区。

   大力推进新型城镇化。加快“三权落实”和“五有并轨”，构建农业转移人口市民化推进机制。推进户籍制度改革，全面实施居住证制度，放开县城和建制镇落户限制，逐步实现基本公共服务由户籍人口向常住人口全覆盖。加强城乡基础设施对接，推进城镇道路建设、污水和垃圾处理、园林绿化、水环境治理，提升县城、重点镇和环湖十二镇的功能和品质，增强对区域经济社会发展的集聚力、辐射力和带动力。坚持市与县（市）联动，拓宽投入渠道，全面推进与国开行合作新型城镇化试点项目建设。

   加快发展县域经济。围绕工业化主战场的定位和跻身全国百强县的目标，以园区为主阵地，全面推动县域工业跨越式发展。加强县域工业园区基础设施建设，提高产业承载力和集聚力。优化园区布局，推动功能相似或相对连片产业功能区整合，建立健全市级以上产业功能区协调发展机制，引导县域经济错位发展。支持肥西桃花工业园区等申报国家级开发区，推动四大开发区与县域工业园区共建一批投资大、就业多、效益好的大项目。继续对巢湖市、庐江县加大政策、资金、人才等支持力度，实现全市县域经济协调发展。

   全面建设美好乡村。大力实施美好乡村“三年行动计划”，完善村庄空间布局，统筹基础设施建设和社会事业发展，削减小型村、空心村和自然村。大力发展城镇交通，改造县乡道路100公里，建设村村通水泥路提级延伸联网工程300公里，实施农村公路危桥改造项目45个。大力推进环巢湖美好乡村示范区建设，重点打造5条示范带和80个重点示范村。大力实施土地整治和城乡用地增减挂钩，探索建立跨区域结对联动机制。

   壮大村级集体经济，实施“十大”到户到人精准扶贫工程。

   （七）大力建设生态文明

   以巢湖生态文明先行示范区建设为统揽，以“水”、“山”、 “气”、“绿”为重点，全面展现碧水青山绿地蓝天的美丽合肥。

   推进水环境治理。加大与国开行合作力度，巩固环巢湖地区生态修复保护一期工程成果，充分发挥效益；全面实施二期工程，确保完成80%工程量；加快推进三期工程，以流域治理、补水引流为重点，突破面源污染治理难点，力争全面开工建设；以开发利用为重点，谋划启动四期工程。积极抓好水生态文明城市试点建设和生态红线划定工作，大力推进南淝河、十五里河和派河综合治理，加快陶冲、蔡田铺二期等污水处理厂建设和提标改造。积极推进“海绵城市”建设，开工一批雨水调蓄池和生态湿地，推进雨水利用，恢复城市生态。全面落实“河长制”，深入实施巢湖流域水污染防治规划，确保达到“十二五”国家考核要求。加大环湖矿山整治和生态修复力度，严厉打击矿山私挖盗采违法行为。

   推进大气污染综合防治。编制大气污染防治项目清单，出台年度考核细则，深入开展“九大行动”，突出抓好“三厂两尘两气”等重点领域专项治理。全部淘汰全社会黄标车，积极推广智能密闭新型渣土车，严格实行午秋两季全市域秸秆禁烧。大力推进“气化合肥”，加快LNG、CNG加气站建设；实施巢湖远景、中国风电等风力发电项目，扩大国家分布式光伏应用示范，逐步优化能源利用结构。加强节能减排，实施一批重点减排项目，深入开展能源等量置换或减量置换，严格控制新增能耗。

   推进国家生态园林城市创建。巩固国家森林城市创建成果，大力实施森林增长工程，完成重点工程造林10万亩、一般成片造林4万亩。高起点规划滁河干渠生态休闲风光带，高标准建设环巢湖公路、高铁南站片区、城市快速路等道路绿化和十五里河等河渠绿化，完成森林长廊156公里。全面开展“三线三边”绿化提升和裸露土地绿化专项治理行动，创建省级森林城市1个、省级森林城镇9个、省级森林村庄50个。扎实推进城镇园林绿化提升，完成城区绿化1000万平方米，打造精品示范工程30个，新建公园游园和街头绿地35个，建设城市绿道90公里以上。依法保护城市绿地、森林、湿地和古树名木，严厉查处损绿毁绿等行为。

   （八）加强社会治理创新

   把保障和改善民生放在突出位置，着力提高社会治理和基本公共服务水平，最大限度地让人民群众共享改革发展成果。

   大力保障改善民生。继续实施民生工程，逐步完善民生工程与社会保障相衔接机制。稳定和扩大就业，提升创业服务水平。加强社会保险体系建设，完善被征地农民基本生活保障制度，逐步扩大失业保险基金支出范围，积极推进各项社会保险关系制度衔接和跨区域转移。新开工各类保障性安居工程2.65万套，新启动城中村和老旧小区改造项目8个、老旧小区环境综合整治项目45个。

   强化基本公共服务。全面实施城市基本公共服务设施专项规划，建立健全以滚动规划为基础、以构建项目库、深化项目前期、衔接部门预算和保障要素供给为主要抓手的公益性项目建设运营新机制。完善社会服务“1+4”政策，加大政府向社会力量购买服务力度。提高普惠性学前教育覆盖率，新建和改扩建公办幼儿园20所。大力推进国家级学校品质提升试验区建设，继续实施义务教育“三大提升工程”，新建中小学15所，建成合肥十中新校区。巩固提升普通高中教育，加大特殊教育扶持力度，加快构建职业教育体系，完善高教城基础设施及配套服务，推动合肥学院、合肥职业技术学院、合肥幼儿师专加快发展。推进省暨合肥市规划馆和省美术馆、百戏城、科技馆等建设，建成市群众文化活动中心。进一步优化卫生资源结构，深化医药卫生改革，促进基层卫生加快发展。加强养老基础设施建设，积极推行“医养结合”的养老新模式，完成50个农村幸福院建设，建成60个社区养老服务站。稳定适度低生育水平，扎实推进“单独二孩”政策。实施全民健身计划，加快市全民健身中心建设，办好环巢湖国际马拉松赛、自行车赛等重大赛事，积极参赛全国首届青年运动会。

   提升社会治理能力。深化法治宣传教育，争创“六五”普法全国先进城市，推动全社会树立法治意识，全面推进法治合肥建设。深化平安合肥建设，着力打造立体化、数字化的社会治安防控体系，依法加强网络社会管理，严厉打击各类违法犯罪活动。保持打击非法传销高压态势。大力推进信访工作改革创新，深入开展矛盾纠纷排查化解，推动联合接访，着力解决疑难复杂信访问题。全面开展民防进社区工作，加强民防指挥基地和应急救援队伍、装备体系建设。严格落实安全生产责任制，大力开展食品药品、消防安全、建筑施工等重点领域专项治理。加强国防和后备力量建设，争创全国双拥模范城“八连冠”。支持工会、共青团、妇联、红十字会等人民团体广泛参与社会管理和公共服务，扎实做好民族宗教、对台侨务、防震减灾、地方志、残疾人、新闻、气象、档案、保密、社科、仲裁等工作。

   三、加强政府自身建设

   站在新起点，面对新任务，我们必须进一步加强政府自身建设，全力打造法治政府。

   （一）坚持依法行政。严格依照法定权限和程序行使权力，自觉接受人大及其常委会依法监督，积极执行人大决议决定，坚持重大决策出台前向人大报告，认真办理人大代表的议案和建议。主动接受政协民主监督，认真办理政协委员提案，广泛听取各民主党派、工商联和无党派人士意见。主动接受司法、舆论、公众监督。完善行政决策机制，建立重大行政决策目录管理制度、终身责任追究制度，落实责任倒查机制。加强重点领域立法，提高立法质量。建立行政执法与刑事司法衔接机制，普遍建立政府法律顾问制度。推进政务公开、办事公开，确保权力在阳光下运行。各级各部门要忠实履行宪法和法律赋予的神圣职责，善于运用法治思维和法治方式推动改革、促进发展、维护稳定。

   （二）坚持为民执政。拓展党的群众路线教育实践活动成果，坚决整治“四风”，完善领导干部直接联系和服务群众机制，多做打基础、利长远的工作，多办顺民心、惠民生的实事，不断提高群众的满意度和幸福感。以推行“三项清单”制度为契机，加快政府职能转变，强化公共服务、市场监管、社会管理、环境保护等职责，维护社会公平正义。完善“12345政府服务直通车”运行机制和政务服务平台软硬件建设，巩固提升政务服务标准化建设成果。各级各部门要牢固树立群众观念和宗旨意识，把更多精力放在制定好政策、提供好服务、营造好环境上，不断创造为民执政的新业绩。

   （三）坚持创新理政。树立全球视野，强化战略思维，在思想观念、体制机制、方式方法上与时俱进，牢牢把握发展大势，切实增强工作主动权。加强新知识学习，加强新政策研究，加强新问题调研，不断提升科学决策水平和驾驭发展能力。依托合肥行政学院等机构，加大公务员队伍培训力度。加强政府智库建设，开展好第三届政府工作创新奖评选，高质量完成“十三五”规划编制。各级各部门要牢固树立创新意识，在解放思想中开拓思路，在见贤思齐中拉高标杆，在大胆探索中破解难题，不断推出更多走在全国前列的“先行之作”。

   （四）坚持务实勤政。自觉践行“三严三实”，严格执行工作责任制、项目负责制、行政问责制，加强对重大决策部署落实情况的督促检查，对确定的目标、制定的政策、部署的任务一抓到底，善做善成。不断改进文风会风，大力精简会议文件，严控各类检查评比。深化行政效能建设，简化办事流程，以铁腕治庸治懒治散，切实解决不作为、慢作为等问题。各级各部门要始终昂扬向上，敢于担当，真抓实干，全面形成心无旁骛抓发展、凝心聚力谋跨越的生动局面。

   （五）坚持廉洁从政。认真履行党风廉政建设主体责任，强化“一岗双责”，完善惩治和预防腐败体系，推进反腐倡廉制度创新，落实重大项目纪检监察派驻制度，加强行政监察和审计监督，坚决查处各类违法违纪案件。全面落实中央“八项规定”、国务院“约法三章”和省市各项规定，厉行节约、反对浪费，严控“三公经费”支出。全体政府工作人员，特别是各级领导干部，要时刻绷紧廉洁自律这根弦，自觉提高拒腐防变和抵御风险能力，努力当好人民公仆。

   各位代表！

   适应新常态任务艰巨，实现新定位使命光荣。让我们紧密团结在以习近平同志为总书记的党中央周围，在省委省政府和市委的坚强领导下，紧紧依靠全市人民，攻坚克难，开拓创新，为建设长三角世界级城市群副中心、打造“大湖名城、创新高地”而努力奋斗！
